# Systematic misclassification of sylvatic dengue virus 2 (DENV-2) infections as “undetermined serotype”: implications in routine RT-qPCR surveillance

**DOI:** 10.1186/s41182-025-00827-0

**Published:** 2025-10-16

**Authors:** Idrissa Dieng

**Affiliations:** 1 Virology Department, Institut Pasteur de Dakar, Dakar, Senegal; 2 Animal Biology Department, Université Cheikh Anta Diop de Dakar, Dakar, Senegal

**Keywords:** Sylvatic DENV-2, RT-qPCR, Misclassified, Undetermined serotype, Surveillance

## Abstract

Recent findings by Suppiah et al. revealed DENV-2 positive samples misclassified as “Undetermined Serotype” when using commercial dengue molecular serotyping assays. This highlights the noninclusion and resulting misclassification of this viral strain as a dengue virus serotype using available molecular tools. Drawing parallels from the notification of the same assay failure against sylvatic DENV-2 strains in Senegal, West Africa, this correspondence advocates for updating the existing tools to account for this viral variant. Development of multiplex assays including both urban and sylvatic strains may enhance dengue virus surveillance and provide real time monitoring of the prevalence of sylvatic viral lineages among recorded dengue infections in surveillance data.

Dear Editor,

We read with great interest the article by Suppiah et al. [1] which provides contemporary evidence of sylvatic DENV-2 re-emergence in Malaysia. This discovery consolidates previous identification of this viral lineage in Senegal (West Africa) in 2020 and 2021 respectively [2, 3]. It also draws attention to a critical but under-recognized limitation in global dengue surveillance: sylvatic dengue virus infections are being systematically reported as “undetermined serotype” based on widely used molecular serotyping assays [1, 4].

Investigations conducted in Kédougou (2020) and Kolda (2021), Senegal, showed that three commonly used RT-qPCR serotyping assays failed to assign a serotype to multiple patient samples that were subsequently confirmed by whole-genome sequencing as sylvatic DENV-2 [2, 4]. Initially logged as assay failures or uninformative results, these samples instead reflected true infections with divergent sylvatic lineages.

Recent surveillance in Malaysia reported repeated “undetermined serotype” results by commercial assays; tiled/shotgun sequencing resolved most as sylvatic DENV-2 and one as a highly divergent DENV-3 lineage [1]. As in Senegal, primer–probe mismatches at several positions in the targets (C-prM/E/NS5 regions, depending on the kit) could explain the false-negative serotyping [4].

Current commercial and laboratory-developed serotyping assays were optimized against urban (human-adapted) DENV1-4 lineages [1]. For sylvatic lineages circulating in nonhuman primates and forest Aedes vectors, accumulated substitutions at primer/probe binding sites reduce effective Tm and binding energy, decreasing amplification efficiency and probe hybridization. The result is nonassignment of the serotype (“undetermined”) despite adequate viral RNA and positive pan-DENV assay results [3].

The public health implications of this are as follows:

- The frequency of sylvatic dengue in humans is likely underestimated, particularly in regions where sylvatic and urban cycles co-occur [5, 6].
- “Undetermined” results can mask early transmission by sylvatic lineages, degrading situational awareness and timeliness of response [7].
- Unrecognized antigenic/genetic diversity may bias assessments of vaccine effectiveness and neutralization breadth if sylvatic lineages circulate undetected [8].

Recommendations for surveillance laboratories and kit developers:

- Treat “undetermined serotype” as a trigger for sequencing (amplicon tiling or metagenomic) and report them as “DENV positive serotype unresolved (sequencing pending)” rather than as an assay failure.
- Mandate routine primer–probe coverage audits against curated panels that include sylvatic reference genomes (West African DENV-2 and other divergent clades). Relevant tools would include thermodynamic mismatch modeling (e.g., ThermoBLAST/MFEprimer), in-silico PCR, and coverage heatmaps.
- Update serotyping assays with degenerate bases or LNA-nucleotidess at documented mismatch positions and consider redundant target regions (e.g., E and NS5) to reduce single-site failure.
- Establish limits of detection (LoD) and ΔCt penalty for representative sylvatic isolates using quantified RNA standards; report cross-reactivity and inclusivity panels that explicitly include sylvatic genomes.
- In surveillance dashboards, separate “undetermined” from “negative” and add a category for “putative sylvatic lineage” before sequencing sequencing confirms divergence.
- Ensure there is routine genomic data flow (sequence and minimal metadata) with rapid lineage assignment and feedback to diagnostic program, prioritizing archiving of all “undetermined” cases.

Convergent observations from Senegal and Malaysia indicate a systemic diagnostic blind spot: current RT-qPCR serotyping assays are narrowly tuned to urban DENV and recurrently miss sylvatic lineages. We advocate urgent assay recalibration and reflex sequencing of “undetermined” results, coupled with primer–probe surveillance against both urban and sylvatic viral diversity. These steps will improve case ascertainment, accelerate outbreak detection, and strengthen vaccine/therapeutic evaluation frameworks.

## Data Availability

No data sets were generated or analyzed during the current study.
